# Characterization of *O*-methyltransferases in the biosynthesis of phenylphenalenone phytoalexins based on the telomere-to-telomere gapless genome of *Musella lasiocarpa*

**DOI:** 10.1093/hr/uhae042

**Published:** 2024-03-08

**Authors:** Wanli Zhao, Junzhi Wu, Mei Tian, Shu Xu, Shuaiya Hu, Zhiyan Wei, Guyin Lin, Liang Tang, Ruiyang Wang, Boya Feng, Bi Wang, Hui Lyu, Christian Paetz, Xu Feng, Jia-Yu Xue, Pirui Li, Yu Chen

**Affiliations:** Jiangsu Key Laboratory for the Research and Utilization of Plant Resources, Jiangsu Province Engineering Research Center of Eco-cultivation and High-value Utilization of Chinese Medicinal Materials, Institute of Botany, Jiangsu Province and Chinese Academy of Sciences (Nanjing Botanical Garden Mem. Sun Yat-Sen), 210014 Nanjing, China; Jiangsu Key Laboratory for the Research and Utilization of Plant Resources, Jiangsu Province Engineering Research Center of Eco-cultivation and High-value Utilization of Chinese Medicinal Materials, Institute of Botany, Jiangsu Province and Chinese Academy of Sciences (Nanjing Botanical Garden Mem. Sun Yat-Sen), 210014 Nanjing, China; Nanjing University of Chinese Medicine, 210023 Nanjing, China; Jiangsu Key Laboratory for the Research and Utilization of Plant Resources, Jiangsu Province Engineering Research Center of Eco-cultivation and High-value Utilization of Chinese Medicinal Materials, Institute of Botany, Jiangsu Province and Chinese Academy of Sciences (Nanjing Botanical Garden Mem. Sun Yat-Sen), 210014 Nanjing, China; Jiangsu Key Laboratory for the Research and Utilization of Plant Resources, Jiangsu Province Engineering Research Center of Eco-cultivation and High-value Utilization of Chinese Medicinal Materials, Institute of Botany, Jiangsu Province and Chinese Academy of Sciences (Nanjing Botanical Garden Mem. Sun Yat-Sen), 210014 Nanjing, China; College of Horticulture, Bioinformatics Center, Academy for Advanced Interdisciplinary Studies, Nanjing Agricultural University, 210095 Nanjing, China; College of Horticulture, Bioinformatics Center, Academy for Advanced Interdisciplinary Studies, Nanjing Agricultural University, 210095 Nanjing, China; Jiangsu Key Laboratory for the Research and Utilization of Plant Resources, Jiangsu Province Engineering Research Center of Eco-cultivation and High-value Utilization of Chinese Medicinal Materials, Institute of Botany, Jiangsu Province and Chinese Academy of Sciences (Nanjing Botanical Garden Mem. Sun Yat-Sen), 210014 Nanjing, China; Jiangsu Key Laboratory for the Research and Utilization of Plant Resources, Jiangsu Province Engineering Research Center of Eco-cultivation and High-value Utilization of Chinese Medicinal Materials, Institute of Botany, Jiangsu Province and Chinese Academy of Sciences (Nanjing Botanical Garden Mem. Sun Yat-Sen), 210014 Nanjing, China; Jiangsu Key Laboratory for the Research and Utilization of Plant Resources, Jiangsu Province Engineering Research Center of Eco-cultivation and High-value Utilization of Chinese Medicinal Materials, Institute of Botany, Jiangsu Province and Chinese Academy of Sciences (Nanjing Botanical Garden Mem. Sun Yat-Sen), 210014 Nanjing, China; Jiangsu Key Laboratory for the Research and Utilization of Plant Resources, Jiangsu Province Engineering Research Center of Eco-cultivation and High-value Utilization of Chinese Medicinal Materials, Institute of Botany, Jiangsu Province and Chinese Academy of Sciences (Nanjing Botanical Garden Mem. Sun Yat-Sen), 210014 Nanjing, China; Jiangsu Key Laboratory for the Research and Utilization of Plant Resources, Jiangsu Province Engineering Research Center of Eco-cultivation and High-value Utilization of Chinese Medicinal Materials, Institute of Botany, Jiangsu Province and Chinese Academy of Sciences (Nanjing Botanical Garden Mem. Sun Yat-Sen), 210014 Nanjing, China; NMR/Biosynthesis Group, Max Planck Institute for Chemical Ecology, Hans-Knöll-Straße 8, 07745 Jena, Germany; NMR/Biosynthesis Group, Max Planck Institute for Chemical Ecology, Hans-Knöll-Straße 8, 07745 Jena, Germany; Jiangsu Key Laboratory for the Research and Utilization of Plant Resources, Jiangsu Province Engineering Research Center of Eco-cultivation and High-value Utilization of Chinese Medicinal Materials, Institute of Botany, Jiangsu Province and Chinese Academy of Sciences (Nanjing Botanical Garden Mem. Sun Yat-Sen), 210014 Nanjing, China; College of Horticulture, Bioinformatics Center, Academy for Advanced Interdisciplinary Studies, Nanjing Agricultural University, 210095 Nanjing, China; Jiangsu Key Laboratory for the Research and Utilization of Plant Resources, Jiangsu Province Engineering Research Center of Eco-cultivation and High-value Utilization of Chinese Medicinal Materials, Institute of Botany, Jiangsu Province and Chinese Academy of Sciences (Nanjing Botanical Garden Mem. Sun Yat-Sen), 210014 Nanjing, China; Jiangsu Key Laboratory for the Research and Utilization of Plant Resources, Jiangsu Province Engineering Research Center of Eco-cultivation and High-value Utilization of Chinese Medicinal Materials, Institute of Botany, Jiangsu Province and Chinese Academy of Sciences (Nanjing Botanical Garden Mem. Sun Yat-Sen), 210014 Nanjing, China

## Abstract

Phenylphenalenones (PhPNs), phytoalexins in wild bananas (Musaceae), are known to act against various pathogens. However, the abundance of PhPNs in many Musaceae plants of economic importance is low. Knowledge of the biosynthesis of PhPNs and the application of biosynthetic approaches to improve their yield is vital for fighting banana diseases. However, the processes of PhPN biosynthesis, especially those involved in methylation modification, remain unclear. *Musella lasiocarpa* is a herbaceous plant belonging to Musaceae, and due to the abundant PhPNs, their biosynthesis in *M. lasiocarpa* has been the subject of much attention. In this study, we assembled a telomere-to-telomere gapless genome of *M. lasiocarpa* as the reference, and further integrated transcriptomic and metabolomic data to mine the candidate genes involved in PhPN biosynthesis. To elucidate the diversity of PhPNs in *M. lasiocarpa*, three screened *O*-methyltransferases (Ml01G0494, Ml04G2958, and Ml08G0855) by phylogenetic and expressional clues were subjected to *in vitro* enzymatic assays. The results show that the three were all novel *O*-methyltransferases involved in the biosynthesis of PhPN phytoalexins, among which Ml08G0855 was proved to function as a multifunctional enzyme targeting multiple hydroxyl groups in PhPN structure. Moreover, we tested the antifungal activity of PhPNs against *Fusarium oxysporum* and found that the methylated modification of PhPNs enhanced their antifungal activity. These findings provide valuable genetic resources in banana breeding and lay a foundation for improving disease resistance through molecular breeding.

## Introduction

Bananas (*Musa* spp.), which originated in Southeast Asia, are one of the most important commercial crops in the world [1]. China is the world’s second-biggest producer of bananas after India, harvesting about 11.7 million tons in 2021 [2]. However, banana yields are severely curtailed by diseases caused by fungi, viruses, and plant-parasitic nematodes. Notably, banana fusarium wilt (BFW), caused by *Fusarium oxysporum* f. sp. *cubense* tropical race 4, is one of cultivated banana’s most destructive diseases; despite decades of research, few effective options for managing this disease have been developed. Planting resistant cultivars is widely considered to be the most important strategy in affected areas [3]. The wild relatives of commercial crops usually possess more genetic diversity and so are often useful for developing more resistant varieties [4], in contrast to cultivated plants, which often produce less variety and/or fewer defense-related secondary metabolites than their wild-type relatives [5, 6]. The use of defense metabolites, e.g. phenylphenalenones (PhPNs), can be an important strategy in overcoming current problems of banana cultivation.

**Figure 1:**
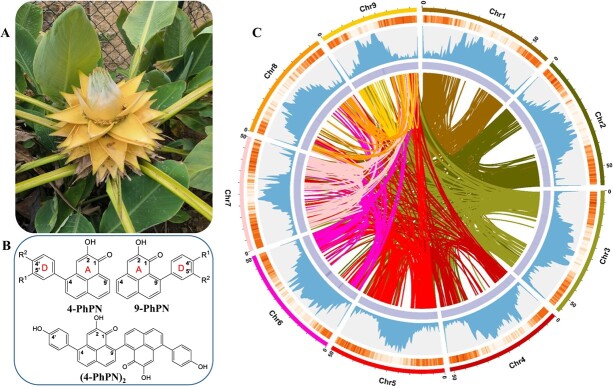
Sequencing samples, overview genome assembly of *M. lasiocarpa*, and PhPN structure. Photograph of *M. lasiocarpa* (**A**), types of PhPNs (**B**; R^1^ and R^2^, -OH or -OCH_3_), and distribution of *M. lasiocarpa* genomic features (**C**). The rings from the outside to the inside indicate nine chromosomes, gene density, repeat density, GC contents, and syntenic genomic blocks in section C.

Phenylphenalenone-type secondary metabolites, which consist of a tricyclic phenalene nucleus and a lateral phenyl ring (Fig. 1B), occur mainly in monocot taxa, like Strelitziaceae and Musaceae [7]. Because PhPNs in wild banana plants were reported to be important phytoalexins and phytoanticipins, they were considered valuable resources for breeding disease-resistant banana cultivars [8, 9]. However, cultivated banana plants contain PhPNs in low concentrations and low structural variety. Genetic engineering could be used to alter that imbalance, influencing the biosynthesis of PhPNs and the enzymes involved in order to tailor disease-resistant plants. Previous studies suggested that PhPNs are biosynthetically derived from the phenylpropanoid pathway and that their linear precursors are transformed through an intramolecular Diels–Alder cyclization [7]. Only a chalcone synthase, *WtPKS1*, catalyzing the first step in diarylheptanoid biosynthesis was characterized from *Wachendorfia thyrsiflora* [10]. All other biosynthetic enzymes that contribute to the formation of PhPNs are still unknown.

*Musella lasiocarpa* is an endemic plant in China and the only member of the genus *Musella* (Fig. 1A). It is mainly distributed in southwestern areas of the country, such as the Yunnan and Sichuan provinces [11, 12]. Because of its long flowering period and beautiful appearance (e.g. large and golden inflorescences), *M. lasiocarpa* is often cultivated as an ornamental plant and is known as Di Yong Jin Lian in Chinese. In addition to its ornamental value, the flowers and bracts of *M. lasiocarpa* are used in traditional folk medicine to stop bleeding and to counteract inflammation [11]. A recent phytochemical investigation of *M. lasiocarpa* showed the plant contained various PhPNs and also linear diarylheptanoids (LDHs), which are considered biosynthetic precursors of PhPNs [13]. Our previous research showed that LDHs and PhPNs accumulate in the seed coats of *M. lasiocarpa* during the middle and late stages of seed development [14], and thus the seeds may be a useful model system for the study of PhPN biosynthesis. In the present work we focused on the *O*-methylation of PhPNs catalyzed by *O*-methyltransferases (OMTs). During *O*-methylation, the transfer of a methyl group from *S*-adenosyl-l-methionine (SAM) to a hydroxyl group of an appropriate substrate is achieved. In plants, *O*-methylation occurs as part of the biosynthesis of many types of secondary metabolites, including terpenoids, alkaloids, flavonoids, and also PhPNs [7, 15–17]. *O*-Methylation of PhPNs contributes significantly to the bioactivity of these metabolites by influencing their diffusion into biological membranes [18]. Previous studies found that methylation of phenolic hydroxyl groups can improve PhPNs’ activity against the pathogen *Mycosphaerella fijiensis* [19].

In this work, we assembled a high-quality genome of *M. lasiocarpa*. Using the genomic data as the fundament, we integrated multiomics data to screen candidate enzymatic genes involved in the methylation of PhPNs, along with functional experiments to verify the real OMT genes and elucidate their diversified functional roles. The present study provides valuable information for the use of genetic resources found in the wild banana relative *M. lasiocarpa* and offers important insights into the function of OMTs in PhPN biosynthesis.

## Results

### Nearly telomere-to-telomere gapless genome assembly and annotation of *M. lasiocarpa*

We sequenced and assembled a telomere-to-telomere gapless genome of *M. lasiocarpa*, based on ~35.49 Gb (>65 X) of HiFi data and 26 Gb (>48 X) of Hi-C data. *K*-mer distribution analysis revealed a genome size of 535 Mb with 1.02% heterozygosity and 51.12% repetition (Supplementary Data Fig. S1), fitting the genome size based on flow cytometry analysis and *k*-mer analysis (Supplementary Data Table S1). The preliminary assembly by HiFi data generated 440 contigs with a total length of 509 Mb and a contig N50 value of 56.62 Mb (Table 1). The completeness of the *M. lasiocarpa* genome assembly was evaluated by BUSCO [20] and CEGMA (https://github.com/marbl/merqury), in which 98.50% complete and 96.77% coverage of the complete matches were identified in the assembly. Then, the *M. lasiocarpa* contigs were further assembled to scaffolds using Hi-C data. Approximately 470 Mb (14 contigs, 92.32%) of the total assembly were successfully anchored into nine pseudochromosomes, corresponding to the nine haplotype chromosomes of *M. lasiocarpa* (Supplementary Data Fig. S2A and Fig. 1C).

**Table 1:** The main genome assembly features of *M. lasiocarpa*.

Assembly feature	Number	Length (Mb)
Total contigs	440	509.15
Contig N50	5	56.62
Contig N90	9	43.00
Total scaffolds	431	509.15
Scaffold N50	5	56.62
Scaffold N90	9	43.04
Pseudochromosomes	9	
Repetitive sequences	51.56%	
Protein-coding genes	34 361	

The identification of centromeres and telomeres indicates that our assembly reached high degrees of completeness and continuity, with all nine centromeres and almost all (16/18) telomeres detected. *Musella lasiocarpa* telomeres consist of tandem repeats of TTTAGGG and are located at both ends of chromosomes, except chromosomes 7 and 9, which only possess an intact telomere at one end. The lengths of the identified *M. lasiocarpa* telomeres vary greatly, ranging from 261 to 5674 bp, and most are >1 kb, with only two below that (Supplementary Data Table S2). The nine *M. lasiocarpa* centromeres consist of tandem repeats of different sequences and varying lengths, and the lengths range from 198 to 2271 kb (Supplementary Data Table S3). These identified telomeres and centromeres make our assembly a telomere-to-telomere gapless genome, with only nine gaps present and one gap for each chromosome. All nine gaps are all found to be located within the telomere regions, so they are unlikely to include any protein-coding genes, which means our current assembly and annotation have covered a complete gene repertoire.

We then performed genome annotation in the *M. lasiocarpa* assembly. A total of 34 361 protein-coding genes were predicted, with an average sequence length of 4379.73 bp per gene, similar to those reported in other plants from the same family (Supplementary Data Table S4) [21–23]. On average, each predicted gene contains 4.92 exons with length 245.10 bp for each exon. Functional annotation captured 97.67% of the protein-coding genes by similarity searches against protein domains and homologous sequences (Supplementary Data Table S5). Moreover, we identified non-coding RNA (ncRNA) genes in the *M. lasiocarpa* assembly, including 12 929 rRNA, 412 miRNA, 380 snRNA, and 3015 tRNA genes (Supplementary Data Table S6).

### Phylogenetic position and genome evolution of *M. lasiocarpa*

**Figure 2:**
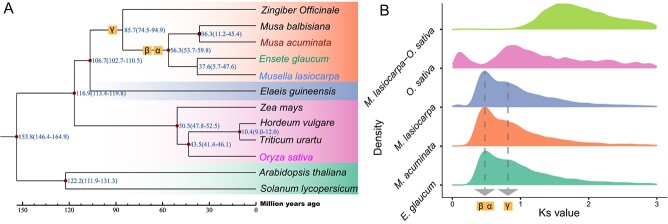
Evolutionary analyses of *M. lasiocarpa*. **A** Phylogeny and divergence time of 12 angiosperms. **B** WGD analysis of four species.

To resolve the phylogenetic position of *M. lasiocarpa* and its relationship with other Musaceae species, a concatenated dataset comprising 1371 single-copy orthologous genes from 12 species was constructed and phylogenomic analysis based on the dataset was performed using the maximum likelihood method. The analysis resolved *M. lasiocarpa* as sister to *Ensete glaucum*, and together they were recovered to be sister to the *Musa* genus. Molecular clock analysis estimated the origin of Musaceae to be around 56.3 million years ago (MYA), and the divergence between *M. lasiocarpa* and *E. glaucum* to be around 37.6 MYA (Fig. 2A). Intergenomic co-linearity analysis (Supplementary Data Fig. S2B) showed an almost one-to-one syntenic relationship at the chromosome level between *M. lasiocarpa* and *E. glaucum*, suggesting a well-preserved genomic structure for the two species. More substantial rearrangement between *M. lasiocarpa* and *Musa acuminata* was also detected. For instance, chromosome 2 of *M. acuminata* was only a part of that of *M. lasiocarpa*, suggesting either a chromosome break event in *M. acuminata* or a chromosome fusion event in *M. lasiocarpa*. By calculating the synonymous mutation rates (*K*_s_) of anchored paralogous gene pairs, we were able to identify potential whole-genome duplications (WGDs) in these species (Fig. 2B). The *K*_s_ density distribution of *M. acuminata* showed two peaks that indicated multiple WGD events, which is in line with *E. glaucum* and banana in the same family [24, 25]. Previous studies reported either three or four rounds of WGDs in the evolution of Musaceae plants, and proposed that the most recent two rounds of WGDs (α and β) occurred consecutively at a similar period around 65 MYA, so that only a single peak was displayed, representing α and β WGDs [24–26]. Our intragenomic synteny analysis found that in the three Musaceae species most paralogous gene clusters shared relationships with three other clusters, with similar *K*_s_ values in *M. lasiocarpa*, *E. glaucum*, and *M. acuminata* (Supplementary Data Fig. S3), supporting the continuous occurrence of WGDs.

### Transcriptional expression of *MlOMT* genes in *M. lasiocarpa* seeds during different developmental stages

**Figure 3:**
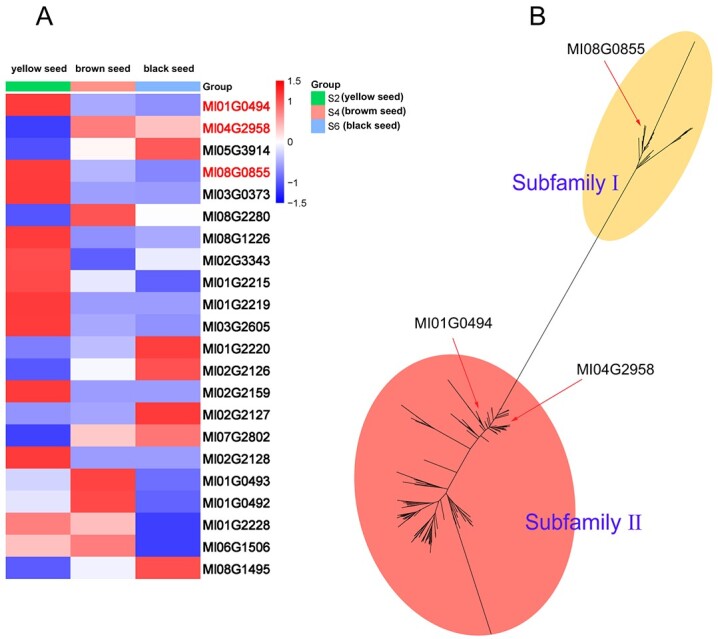
Expression heat map and phylogeny of *MlOMT* genes. **A** Expression of 30 *MlOMT* genes in *M. lasiocarpa* seeds during three developmental stages (yellow seed, S2; brown seed, S4 and black seed, S6) based on the transcriptomic data. The FPKM values of Ml01G0494, Ml04G2958, and Ml08G0855 (marked in red) were greater than 100. The expression level was measured by FPKM. Eight genes (Ml01G2217, Ml01G2223, Ml01G2225, Ml01G2224, Ml02G2158, Ml01G1752, Ml01G1753, and Ml06G1505) that were not expressed in all samples are not listed. **B** Phylogeny of OMTs from 12 genomes and characterized enzymes.

Methyltransferases, a subclass of the transferase family, plays a vital role in the formation of secondary metabolites in plants. Methyltransferases usually have conserved substrate-binding domains and methyl donor-binding domains. We searched for candidate methyltransferase genes (*MlOMT*s) by combining homology-based BLAST and conserved domains (PF01596 or PF00891) in our *M. lasiocarpa* genome database [27–29]. In total, 30 *MlOMT* genes were predicted in the *M. lasiocarpa* genome (Supplementary Data Fig. S4). Next, we compared and analyzed the PhPN components in *Musa* and their related plant species. Results showed that PhPNs were more abundant in *M. lasiocarpa* than in other banana species. Especially in mature seeds of *M. lasiocarpa* the content of PhPNs was high. We then analyzed the content of PhPNs in seeds of *M. lasiocarpa* at three developmental stages (S2, yellow seed; S4, brown seed; and S6, black seed) by high-performance liquid chromatography/quadrupole time-of-flight mass spectrometry (HPLC/Q-TOF–MS). The results showed that the PhPN content increased gradually in the order S2 < S4 < S6 (Supplementary Data Fig. S5). The results showed that the distribution and accumulation of PhPNs were time-specific in seeds of different stages. This was in accordance with previously published results [14]. We investigated the expression of *MlOMT* genes in *M. lasiocarpa* seeds of different developmental stages. Most *MlOMT*s were unexpressed or expressed only at low levels. Only three *MlOMT* genes (Ml01G0494, Ml04G2958, and Ml08G0855, FPKM values ≥100) were more highly expressed (Fig. 3A). Furthermore, Ml01G0494, Ml04G2958, and Ml08G0855 were stage-specifically expressed. The expression level of Ml01G0494 was 5.98-fold higher in S2 than in S6, while Ml04G2958 was expressed 2.21-fold lower in S2 than in S6 (Supplementary Data Table S7). Therefore, Ml01G0494, Ml04G2958, and Ml08G0855 were selected as candidate genes that were probably involved in the biosynthesis of methoxylated PhPN.

We gathered all OMTs identified from 12 plant genomes (the same species used for species evolutionary analysis in Fig. 2A) and from other plants previously characterized, and performed phylogenetic analysis for the OMTs. It turned out that all OMTs should be classified into two subfamilies, namely Methyltransf_24 (subfamily I) and Methyltransf_2 (subfamily II) (Fig. 3B and Supplementary Data Fig. S6). In fact, the two subfamilies showed very low sequence identity. To be precise, they should be considered as two gene families with functional convergence. Of our three screened genes, Ml01G0494 and Ml04G2958 fell into Methyltransf_2 and Ml08G0855 fell into Methyltransf_24, suggesting the convergent evolution of their potential functions.

### Functional characterization and subcellular localization analysis of MlOMTs

**Figure 4:**
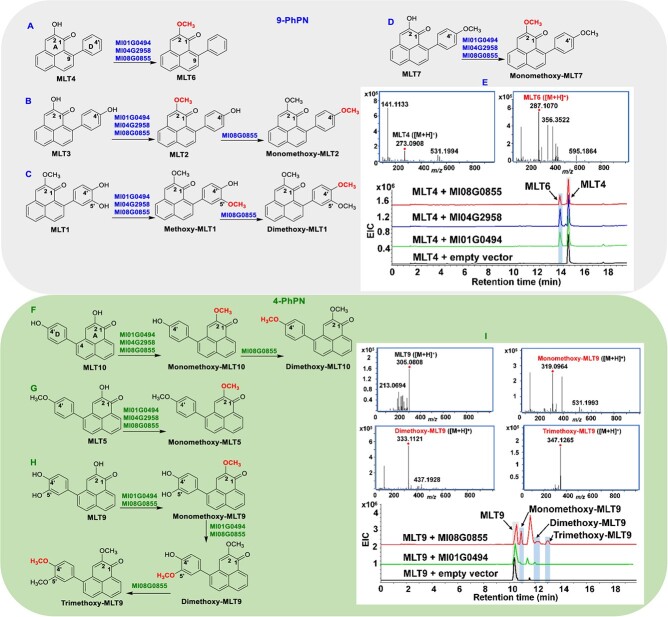
Results of MlOMT assays with PhPNs. **A**–**D** and **F**–**H** Schematic diagram of catalytic reaction between MlOMTs and PhPNs. **E**, **I** HPLC/Q-TOF–MS results of representative substrates MLT4 and MLT9 reacted with Ml01G0494, Ml04G2958, and Ml08G0855.

The three candidate *MlOMT* genes, Ml01G0494, Ml04G2958, and Ml08G0855, were cloned into pMAL-c4x vectors with MBP tags and subsequently expressed in *Escherichia coli* BL21 (DE3) strain. The purified bands of the candidate MlOMT proteins were analyzed by SDS–PAGE and found to be in accordance with theoretical molecular weights (Supplementary Data Fig. S7). To characterize the catalytic activity of the putative MlOMTs Ml01G0494, Ml04G2958, and Ml08G0855 *in vitro*, PhPN substrates with variable degrees of hydroxylation in rings A and/or D were used for methylation assays (Fig. 4). We examined three types of PhPNs, 4-PhPNs, 9-PhPNs, and a dimeric 4-PhPN (Fig. 1B). Crude MlOMT proteins were used for the assays, and the reaction products were analyzed by HPLC/Q-TOF–MS. The results revealed that the MlOMTs exhibited differential catalytic activity on the A or D ring of the PhPNs with regioselectivity (Fig. 4, Supplementary Data Fig. S8, and Supplementary Data Table S8). When the enzymes were tested with 9-PhPNs, the *O*-methylation of position 2 of ring A (Fig. 4A, B, D, and E) was observed. When the 9-PhPN was methoxylated in position 2, and hydroxylation of positions 4′ and 5′ of ring D was present, a monomethylation of position 5′ was observed (Fig. 4C). Ml08G0855 was able to further methylate the reaction products MLT2 (Fig. 4B) and methoxy-MLT1 (Fig. 4C). The dimeric compound (4-PhPN)_2_ (Fig. 1B) could not be methylated by any of the enzymes. Compounds of this type seem too bulky to be suitable substrates for the enzymes.

In the next series of assays 4-PhPNs were examined. The methylation of position 2 could be observed by all three enzymes with a substrate as depicted in Fig. 4F, G, and H. Again, Ml08G0855 was able to methylate hydroxy functions in ring D. When monomethoxy-MLT10 was used as substrate, it was translated into dimethoxy-MLT10 by this enzyme (Fig. 4F). We used the observed regiospecificities in an experiment that resulted in the sequential methylation of all hydroxy groups in a 4-PhPN substrate. In the first step, a substrate methoxylated in position 2 was recovered from the assay when Ml01G0494 or Ml08G0855 was used with a triply hydroxylated (positions 2, 4′, and 5′) substrate (Fig. 4H and I). The reaction product was then further incubated with Ml01G0494 or Ml08G0855 to achieve monomethoxylation of position 5′. The triple-methoxylation of position 4′ was only produced by Ml08G0855. Results of this experiment show that Ml08G0855 is capable of catalyzing the methylation of a wide array of hydroxylated PhPNs.

**Figure 5:**
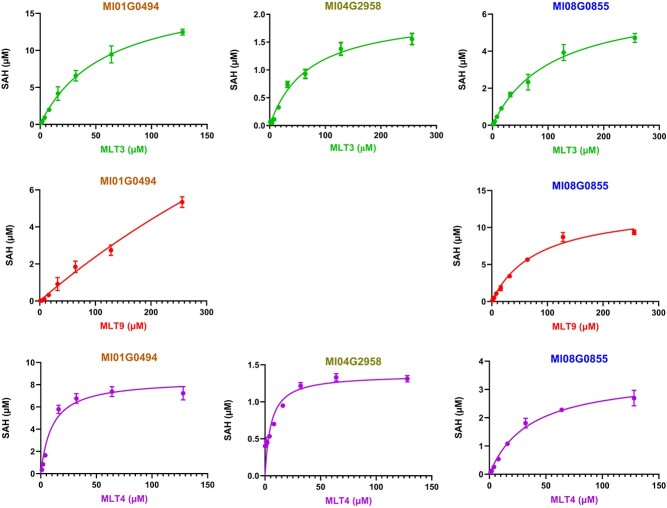
Kinetic properties of recombinant MlOMTs with different PhPN substrates. Kinetic parameters were estimated by non-linear curve fitting using the Michaelis–Menten tool. The concentration of SAH generated during the substrate reaction was assayed.

In order to optimize the optimal reaction conditions of MlOMT recombinases, the pH and temperature for the *in vitro* catalytic reaction of MlOMT recombinases were examined using MLT4 as a substrate. Results showed that MlOMTs displayed optimal activity in 50 mM Tris–HCl buffer (pH 8.0) at 45°C (Supplementary Data Fig. S9). *K*_m_ and *K*_cat_ values were calculated by non-linear curve fitting using the Michaelis–Menten model (Fig. 5). As illustrated in Supplementary Data Table S9, the apparent *K*_m_ value of Ml01G0494 for MLT9 (970.60 μM) was the highest; however, the *K*_cat_/*K*_m_ value of Ml01G0494 for MLT9 (2.19 μM^−1^ s^−1^) was the lowest. The apparent *K*_m_ and *K*_cat_/*K*_m_ values of Ml08G0855 for MLT9 were 81.93 μM and 12.24 μM^−1^ s^−1^. The above results indicated that the catalytic efficiency of Ml08G0855 for substrate MLT9 was better than that of Ml01G0494. The apparent *K*_cat_/*K*_m_ values of Ml01G0494, Ml04G2958, and Ml08G0855 for MLT3 were 25.58, 7.14, and 8.88 μM^−1^ s^−1^, respectively. Similarly, the apparent *K*_cat_/*K*_m_ values of Ml01G0494, Ml04G2958, and Ml08G0855 for MLT4 were 84.24, 63.24, and 7.42 μM^−1^ s^−1^, respectively. Overall, Ml01G0494 was the more efficient enzyme for the methylation of MLT3 and MLT4 compared with Ml04G2958 and Ml08G0855, while for substrate MLT9 Ml08G0855 was more efficient than Ml01G0494.

To analyze the subcellular localization of MlOMTs, recombinant Ml01G0494, Ml04G2958, and Ml08G0855 plasmids fused with GFP were transiently expressed in *Nicotiana benthamiana* leaves (the primers are listed in Supplementary Data Table S10). As shown in Supplementary Data Fig. S10, the fluorescent signals of the three MlOMTs’ fusion GFP proteins were distributed throughout the cytoplasm and the nucleus. Such a pattern indicated that the cytoplasm might be the subcellular site for the PhPN *O*-methylation.

### Antifungal activity of PhPNs against *Fusarium oxysporum*

To investigate their antifungal activity, PhPNs were tested against the banana pathogen *F. oxysporum* f. sp. *cubense*, Foc 4, the cause of the devastating Panama disease. MLT1, MLT3, MLT4, MLT6, MLT7, MLT9, MLT10, and MLT11 exhibited significant inhibitory activity against the pathogen (Supplementary Data Figs S11 and S12). In particular, methylated products from the assays shown in Fig. 4A and H and the substrate from Fig. 4C and H showed antifungal activity that outcompeted the commercial fungicide, thiophanate methyl (TM). Generally, when 2-hydroxyl groups were derivatized by methylation in either 4- or 9-PhPNs, antifungal activities were enhanced. Clearly, *O*-methylated modifications of PhPNs possess effective antifungal activities.

## Discussion

Bananas are a staple food for millions of people in the tropics and subtropics, but the yield and quality of the fruits are increasingly affected by diseases as global warming progresses. However, effective approaches to control these diseases other than the increased use of pesticides are so far lacking [30]. Conventional pesticides may cause environmental contamination and also affect food safety. Because of their low concentrations in cultivated varieties of sweet banana, PhPNs cannot act as endogenous defensive substances. Mainly found in the plant families Musaceae, Strelitziaceae, and Pontederiaceae [14, 31, 32], PhPNs have recently been isolated and characterized. Once the biosynthetic pathway leading to PhPNs was identified, modern methods of plant breeding by genetic engineering may be used to increase the concentration of PhPNs in commercial banana crops. The wild banana relative we chose for our study (*M. lasiocarpa*) has a higher PhPN content than that of commercially cultivated banana. To further explore the biosynthetic pathway of PhPNs, we assembled a chromosome-level genome assembly of *M. lasiocarpa* obtained by combining PacBio, Bionano, Hi-C, and Illumina sequencing technology. At present, genome sequencing has been conducted on a variety of plants of the Musaceae family, such as *M. acuminata*, *Musa balbisiana*, *E. glaucum*, and *Musa beccarii* [21, 22, 24, 25]. However, the genome data of species with high PhPN content was missing. We sequenced the only member of the genus *Musella*, which laid the foundation for mining PhPN biosynthetic genes. We then analyzed the metabolism of PhPNs in seeds of *M. lasiocarpa* at developmental stages and combined our results with data from the transcriptome analysis. This led us to the discovery of several OMT genes putatively involved in the biosynthesis of PhPNs. After heterologous expression, the resulting enzymes were tested regarding their catalytic activity in *in vitro* assays and the reasons for the diversity of methylated PhPNs were identified. Our OMTs show strong substrate specificity leading to specific methoxylation patterns. This finding firmly corroborates earlier studies that identified *O*-methylation occurring at the end of PhPN biosynthesis [18]. Methylation is an important modification of plant secondary metabolites and can change their physical and chemical properties, including stability and solubility [33]. Previous studies have shown that the 4′-hydroxymethylation of PhPNs could improve inhibitory activity against *F. oxysporum*, the pathogen causing banana fusarium wilt [34]. Furthermore, the 2,4-dimethoxyphenyl groups on the PhPN skeleton increased the antifungal activity against *F. oxysporum* of the skeleton [35]. Finally, we verified the *in vitro* activity of PhPNs and found that the methylated products synthesized by OMTs possess significantly increased antifungal activity. Our results are consistent with previous studies suggesting that methylated PhPN could significantly affect their biological activities. The present study confirms the beneficial function of the identified genes. By introducing them into existing banana breeds they can be used to fight diseases.

## Materials and methods

### Plant materials

For genomic studies, yellow-bracted *M. lasiocarpa* were collected in Nanhua County, Yunnan Province, China (118°50′38″ E, 32°3′44″ N) and transplanted in the experimental field, Nanjing, China (101°1′2″ E, 25°9′54″ N). For RNA sequencing, the leaves, stems, and seeds at three developmental stages were collected (S2, S4, and S6) from the same individual plant.

### Whole-genome sequencing

Young leaves of *M. lasiocarpa* were used to extract genomic DNA by the Plant DNA Kit (Tiangen, China). DNA quality was assessed using NanoDrop 2100 spectrophotometry (Agilent, USA) and agarose gel electrophoresis, followed by Qubit fluorometry (Thermo Fisher Scientific, USA). An Illumina HiSeq X Ten platform was used to construct and sequence the short paired-end libraries. For long-read sequencing, the PacBio library was constructed and sequenced on the PacBio Sequel II platform. The 15-kb preparation solutions were used to construct a SMRTbell target size library. The obtained genomic DNA was cross-linked, subjected to restriction enzyme digestion, and labeled via biotinylated residues for Hi-C sequencing. Biotinylated constructs were enriched, sheared, and sequenced by the Illumina HiSeq X Ten platform. *K*-mer analysis is widely used in genome size evaluation. *K*-mers of 17–31 bp were counted via Jellyfish (version 2.2.7) and the GenomeScope website was used for estimating genome size and heterozygosity according to *k*-mer frequency.

### RNA sequencing

*Musella lasiocarpa* seeds from three developmental stages (S2, S4, and S6) were selected for transcriptome sequencing. These samples were collected from *M. lasiocarpa* and frozen in liquid nitrogen after incubation in RNAlater. Total RNA of each sample was extracted using a plant RNA isolation kit (RC411, Vazyme, Nanjing, China). The mRNA sequencing library was constructed and then sequenced on the Illumina Novaseq 6000 platform. For full-length transcriptome sequencing, mixed RNA library from leaves, seeds, and roots of *M. lasiocarpa* was sequenced by PacBio Sequel II.

### Genome assembly and annotation

To construct the *M. lasiocarpa* genome, hifiasm (v0.16.1) was used to generate assembly contigs with HiFi reads [36]. Then, the draft genome was assembled into scaffolds with Hi-C data by the 3D-DNA pipeline tool [37]. These scaffolds were roughly split via the Juicebox tool and another round of scaffolding. The completeness of genome assembly was assessed by BUSCO [20] and transcriptome data. Homology-based and *ab initio* prediction approaches were used for repeat analysis, and RepeatMasker was applied to identify homologous sequences according to the RepBase (v21.12) library [38]. The data from *ab initio*, homology-based, and transcriptome data evidence approaches were then combined for gene structure annotation. For the RNA sequencing used in the genome annotation, RNA sequencing reads were mapped to the genome using the HISAT2 (v_2.1) program, and the transcripts were assembled via Cufflinks software [39]. High-confidence gene models for the *M. lasiocarpa* genome were predicted by the MAKER pipeline tool. Protein functional annotation was performed by eggNOG-mapper and compared using BLASTp with data stored in the KEGG, DOG, GO, NR, and Swiss-Prot databases. Potential telomeres and centromeres were identified using TeloExplorer and CentroMiner integrated into quarTeT [40].

### Gene family and genome evolution analysis

Protein sequences in the genome are filtered by retaining the longest isomers and discarding sequences with fewer than 50 amino acids. Then, an all-against-all comparison via BLASTp with an E-value cutoff of 1e10−5 was performed [41], and OrthoMCL (http://orthomcl.org/orthomcl/) was applied to cluster genes from these different species into gene families [42]. Expansions and contractions of orthologous groups were identified with Cafe (v4.2, http://sourceforge.net/projects/cafehahnlab/). MCMC tree from PAML (v4.9j) was used for estimating species divergence times. In order to identify genome synteny, the synteny blocks within the *M. lasiocarpa* genome and other species were identified by MCScanX. WGD analysis was conducted using wgd [43].

### HPLC/Q-TOF–MS analysis of phenylphenalenones

Samples of *M. lasiocarpa* seeds of different development stages were ground to powder prior to extraction. To 50 mg of ground sample was added 1 ml methanol and extraction was conducted for 35 min in an ultrasonic bath. The mixture was centrifuged for 10 min at 12 000 rpm. After centrifugation (12 000 rpm, 10 min) the supernatant was subjected to analysis, which was accomplished with HPLC/Q-TOF–MS (Agilent, USA). Chromatography was carried out using an Agilent Poroshell C_18_ column (4.6 mm × 100 mm length, 2.7 μm pore size). The column oven temperature was set to 35°C. The parameters of gradient elution were set as follows: phase A (water with 1‰ formic acid); phase B (methanol): 5–100% B at 0–60 min, 100% B at 60–70 min, 100–95% B at 70–71 min, and 95% B at 71–90 min. The injection volume and flow rate were kept at10 μl and 1 ml/min, respectively. Mass spectra were acquired using electrospray ionization (ESI) in the positive mode. The parameters of the ESI source were set as follows: 10.0 l/min drying gas (N_2_) flow; capillary voltage was 4.0 kV; temperature was 350°C; fragmentation voltage was 170 V; and nebulizer pressure was set to 50 psig. Mass spectral data were acquired in a scanning range from *m*/*z* 100 to 2000. For data collection and instrument control, Qualitative Analysis B.05.00 software was applied.

### Identification and characterization of methyltransferases

A combined methodology including Pfam searching and homologous alignment was used for discovering the methyltransferase genes in the genome of *M. lasiocarpa*. Two conserved domains, PF01596 and PF00891, were used for a genome-wide search by HMMER v_3.3 and BLAST by an e-value of 1e−5. The search was performed to compare the presence of homologs with library entries. Recombinant plasmids were constructed similarly to a previously reported method with minor modifications [44]. The coding DNA sequences of *MlOMT* candidate genes (Supplementary Data Table S11) were cloned into pMAL-c4x (EcoRI/SalI) to yield recombinant plasmids. The recombinant plasmids were further identified by Sanger sequencing, and they were then introduced into BL21 (DE3) for recombinant protein expression. Engineered strains of *E. coli* harboring recombinant plasmids were cultured in 50 ml lysogeny broth (LB) medium at 37°C for 3 h, then induced by 0.1 mM IPTG, followed by a further incubation at 16°C for 24 h. The cells were harvested via centrifugation (6000 rpm, 4°C, and 3 min) and the pellet was resuspended in binding buffer (100 mM Tris–HCl under pH 7.5). The suspension was homogenized in an ultrasound bath for 20 min. Cell debris was subsequently removed by centrifugation at 6000 rpm for 10 min. The supernatant was collected and purified with an ÄKTA protein purification device. The relative activities of the crude recombinant enzymes were then measured. The reaction mixtures (100-μl batches) contained 100 mM Tris–HCl buffer, 10% glycerol, 1 mM β-mercaptoethanol, 2 mM SAM, and the respective crude enzyme. Heat-inactivated enzymes (inactivated at 100°C for 10 min) were used as negative controls. The assay products were analyzed using HPLC/Q-TOF–MS. Enzyme assays determining activities of MlOMTs were measured using the MTase-Glo™ methyltransferase assay according to previously published reports [45]. For example, a series of PhPNs along with the purified recombinant enzymes and 2 mM SAM were used to conduct kinetic analyses during incubation at 45°C for 30 min. The reaction was quenched by adding 0.5% trifluoroacetic acid, followed by determination of the produced *S*-adenosyl-L-homocysteine (SAH) by means of luminescence measurements. The kinetic parameters of *K*_m_ and *K*_cat_ were calculated via the Michaelis–Menten tool implemented in GraphPad Prism (v5).

### Subcellular localization analysis

*MlOMT* genes were cloned into the expression vector (pBinPLUS.GFP4, with a CaMV 35S promoter and GFP), and the resulting recombinant plasmid was transferred into *Agrobacterium* MSU440 via the conventional freeze–thaw method. Next, the empty pBin-GFP and pBin-MlOMTs-GFP MSU440 plasmids were suspended in expression buffer (10 mM MES, 100 μM acetosyringone, and 10 mM MgCl_2_) and subsequently infiltrated into *N. benthamiana* leaves (4 weeks old). After the infiltrated *N. benthamiana* plants had been kept in darkness for 48 h, leaf samples were collected and examined using a laser scanning confocal microscope.

## Acknowledgements

This work was supported by the National Natural Science Foundation of China (grants 32070360, 32200326, and 22207047), the Natural Science Foundation of Jiangsu Province (BK20220752 and BK20220749), and the Jiangsu Institute of Botany Talent Fund (JIBTF202304). We thank Professor Zefu Wang (Nanjing Forestry University) for critical reading of the manuscript and Emily Wheeler for editorial assistance.

## Author contributions

Y.C. and P.R.L. designed the experiments. W.L.Z. and J.Z.W. performed the experiments. M.T., S.Y.H., Z.Y.W., and G.Y.L. performed genomic analysis. S.X. and B.W. carried out antibacterial assays. L.T., R.Y.W., and B.Y.F. isolated and characterized the compounds. W.L.Z., J.Y.X., and Y.C. wrote the paper. C.P., H.L., and X.F. reviewed and revised the paper. All authors contributed to discussion of the manuscript.

## Data availability

Reference genome data and transcriptome sequence reads are available in GenBank under project number PRJNA1009687.

## Conflict of interest

The authors declare that there are no conflicts of interest.

## Supplementary data

Supplementary data are available at *Horticulture Research* online.

## Supplementary Material

Web_Material_uhae042
