# Supplementary material for: Characterization of O-methyltransferases in the biosynthesis of phenylphenalenone phytoalexins based on the telomere-to-telomere gapless genome of Musella lasiocarpa
Source: Hortic Res. 2024 Mar 8;11(4):uhae042. doi: 10.1093/hr/uhae042 (PMC11528125; doi:10.1093/hr/uhae042)
Supplement: Web_Material_uhae042 [file web_material_uhae042.zip › Supporting Information-ML-20240120-added Figure S6.pdf]

Supporting Information

Table S1. Nuclear DNA contents of *Musella lasiocarpa* obtained from flow cytometry analyses

Name	Rf species	Rf Fluorescence	Ml Fluorescence	Genome (Mbp)	Average (Mbp)
Musella lasiocarpa	*Solanum lycopersicum*	25.26	16.04	571.5	566.03 ± 4.79
		25.34	15.88	564.01	
		25.26	15.79	562.59	

Table S2. Results of the identified *M. lasiocarpa* telomeres

Chr id	Chr len	status	Left num	Left direction	Right num	Right direction
Chr1	59609132	both	5540	+	1333	-
Chr2	58485594	both	5674	+	1813	-
Chr3	56666846	both	1131	+	2061	-
Chr4	51710631	both	2723	+	261	-
Chr5	50690933	both	1294	+	1374	-
Chr6	50658751	both	2032	+	1862	-
Chr7	50153042	right	0	none	5078	-
Chr8	49079547	both	1600	+	542	+
Chr9	43037276	left	2242	+	0	none

Table S3. Results of the nine *M. lasiocarpa* centromeres

Chr	start	end	length	TR length	TR coverage
Chr1	25845470	33130712	7285243	1709552	23.47%
Chr2	26084620	32270233	6185614	1835386	29.67%
Chr3	28704488	32155210	3450723	2271236	65.82%
Chr4	25021234	32874427	7853194	2158900	27.49%
Chr5	21982746	23366672	1383927	198521	14.34%
Chr6	29504055	31287580	1783526	926268	51.93%
Chr7	21579267	25074025	3494759	1468809	42.03%
Chr8	16135682	17900334	1764653	861215	48.80%
Chr9	19782222	22709292	2927071	1918863	65.56%

Table S4. The structure genes of *M. lasiocarpa* and other species.

Species	Number	Average transcript length (bp)	Average CDS length (bp)	Average exons per gene	Average exon length (bp)	Average intron length (bp)
Ml	34361	4379.73	1206.77	4.92	245.10	808.70
Nip	35281	2215.86	1013.48	3.85	263.27	421.96
Egla	36835	3929.91	1114.39	4.86	229.09	728.58
Zoff	68096	4993.24	1274.72	5.19	245.81	888.36
Gmax	55702	3142.74	1172.04	5.06	231.74	485.69
Mbal	35111	4443.67	1145.00	5.06	226.37	812.88
Macu	30644	4440.22	1303.02	5.36	242.88	718.73
Atha	27310	1887.74	1229.90	5.16	238.41	158.18

Note: Atha, *Arabidopsis thaliana*; Egla, *Ensete glaucum*; Gmax, *Glycine max*; Macu, *Musa acuminata*; Mbal, *Musa balbisiana*; Nip, *Oryza sativa*; Zoff, *Zingiber officinale*.

Table S5. Gene function annotation results of *M. lasiocarpa*.

Ml	Number	Percent (%)
Total	34361	-
Swissprot	26548	77.26
Nr	32990	96.01
KEGG	25063	72.94
InterPro	32629	94.96
GO	19911	57.95
Pfam	25677	74.73
Annotated	33562	97.67
Unannotated	799	2.33

Table S6. The statistical results of non-coding RNA in *M. lasiocarpa* genome

Type		Copy number	Average length (bp)	Total length (bp)	Percent of genome (%)
miRNA		412	126.75	52220	0.010256
tRNA		3015	75.65	228086	0.044794
rRNA	rRNA	12929	334.66	4326755	0.85
	18S	1654	1724.48	2852289	0.56
	28S	6308	144.13	909193	0.18
	5.8S	1580	158.66	250677	0.049231
	5S	3387	92.88	314596	0.061784
snRNA	snRNA	380	120.65	45848	0.009004
	CD-box	197	104.56	20598	0.004045
	HACA-box	55	138.09	7595	0.001492
	splicing	127	136.48	17333	0.003404
	scaRNA	1	322	322	0.000063
	Unknown	0	0	0	0

Table S7. Relative expression levels of MlOMT genes measured by FPKM

Gene ID	S2	S4	S6
Ml01G0494	4355.99	1029.80	728.66
Ml04G2958	55.68	140.88	123.20
Ml05G3914	50.24	59.37	67.17
Ml08G0855	475.54	188.93	140.17
Ml03G0373	1.43	0.06	0.04
Ml08G2280	4.62	6.87	5.72
Ml08G1226	2.40	0.25	0.42
Ml02G3343	1.52	1.06	1.25
Ml01G2215	0.08	0.03	0.00
Ml01G2219	0.03	0.00	0.00
Ml03G2605	1.59	0.33	0.24
Ml01G2220	0.00	0.04	0.18
Ml02G2126	0.02	1.33	2.82
Ml02G2159	0.07	0.00	0.00
Ml02G2127	0.02	0.06	0.66
Ml07G2802	3.72	5.25	5.76
Ml02G2128	7.09	0.00	0.00
Ml01G0493	6.46	9.07	5.32
Ml01G0492	3.96	5.58	2.94
Ml01G2228	0.14	0.13	0.06
Ml06G1506	0.03	0.03	0.00
Ml08G1495	0.45	2.85	5.85

Table S8. The result of Ml01G0494, Ml04G2958, and Ml08G0855 recombinant enzyme catalyzed products by HPLC/Q-TOF MS

Sample	t_R (min)	Molecular Formula	$[M+H]^+$	Error (ppm)	Identification
MLT1 + empty vector	10.58	$C_{20}H_{14}O_4$	319.95	4.67	MLT-1
MLT1 + Ml01G0494	11.64	$C_{22}H_{18}O_4$	333.1118	1.01	Methoxy-MLT1
MLT1 + Ml08G0855	11.52	$C_{21}H_{16}O_4$	333.1105	4.92	Methoxy-MLT1
MLT1 + Ml08G0855	12.93	$C_{21}H_{16}O_4$	347.1292	-4.09	Dimethoxy-MLT1
MLT2 + empty vector	11.53	$C_{20}H_{14}O_3$	303.1018	-0.76	MLT2
MLT3 + empty vector	12.11	$C_{19}H_{12}O_3$	289.0861	-0.62	MLT3
MLT3 + Ml01G0494	11.52	$C_{20}H_{14}O_3$	303.1015	0.23	MLT2
MLT3 + Ml04G2958	11.53	$C_{20}H_{14}O_3$	303.1029	-4.4	MLT2
MLT3 + Ml08G0855	11.51	$C_{20}H_{14}O_3$	303.096	6.52	MLT2
MLT3 + Ml08G0855	12.6	$C_{21}H_{16}O_3$	303.1005	3.54	Monomethoxy-MLT2
MLT4 + empty vector	14.94	$C_{19}H_{12}O_2$	273.0908	0.76	MLT4
MLT4 + Ml01G0494	14.24	$C_{20}H_{14}O_2$	287.1075	-2.95	MLT6
MLT4 + Ml04G2958	14.24	$C_{20}H_{14}O_2$	287.1078	-4	MLT6
MLT4 + Ml08G0855	14.24	$C_{20}H_{14}O_2$	287.107	-2	MLT6
MLT5 + empty vector	14.7	$C_{20}H_{14}O_3$	303.1008	2.55	MLT5
MLT5 + Ml01G0494	14.43	$C_{21}H_{16}O_3$	317.1162	3.23	Methoxy-MLT5
MLT5 + Ml04G2958	14.49	$C_{21}H_{16}O_3$	317.1159	4.18	Methoxy-MLT5
MLT5 + Ml08G0855	14.46	$C_{21}H_{16}O_3$	317.1164	2.6	Methoxy-MLT5
MLT7 + empty vector	14.71	$C_{20}H_{14}O_3$	303.1005	3.54	MLT7
MLT7 + Ml01G0494	14.06	$C_{21}H_{16}O_3$	317.1154	5.76	Methoxy-MLT7
MLT7 + Ml04G2958	14.1	$C_{21}H_{16}O_3$	317.1145	8.61	Methoxy-MLT7
MLT7+ Ml08G0855	14.06	$C_{21}H_{16}O_3$	317.1148	7.66	Methoxy-MLT7
MLT9 + empty vector	10.7	$C_{19}H_{12}O_4$	305.0809	-0.21	MLT9
MLT9 + Ml01G0494	11.08	$C_{20}H_{14}O_4$	319.0964	0.27	Monomethoxy-MLT9
MLT9 + Ml01G0494	12.53	$C_{21}H_{16}O_4$	333.1126	-1.4	Dimethoxy-MLT9
MLT9+ Ml08G0855	11.06	$C_{20}H_{14}O_4$	319.0964	0.27	Monomethoxy-MLT9
MLT9+ Ml08G0855	12.5	$C_{21}H_{16}O_4$	333.1121	0.11	Dimethoxy-MLT9
MLT9+ Ml08G0855	12.94	$C_{22}H_{18}O_4$	347.1278	3.71	Trimethoxy-MLT9
MLT10 + empty vector	11.66	$C_{19}H_{12}O_3$	289.0856	1.11	MLT10
MLT10 + Ml01G0494	11.4	$C_{20}H_{14}O_3$	303.1014	0.57	Monomethoxy-MLT10
MLT10 + Ml04G2958	11.42	$C_{20}H_{14}O_3$	303.1014	1.56	Monomethoxy-MLT10
MLT10 + Ml08G0855	12.44	$C_{21}H_{16}O_3$	319.0964	2.28	Dimethoxy-MLT10

Table S9. Kinetic properties of MlOMTs with different PhPN substrates.

	MLT3			MLT4			MLT9	
	Ml01G0494	Ml04G2958	Ml08G0855	Ml01G0494	Ml04G2958	Ml08G0855	Ml01G0494	Ml08G0855
Km (μM)	60.43±16.63	69.94±14.71	110.90±27.41	8.51±0.13	5.30±0.68	37.32±10.68	970.60±227.70	81.93±7.04
Vmax (μM/min)	0.61	0.07	0.23	0.28	0.05	0.12	0.84	0.43
Kcat ($10^{-3}s^{-1}$)	1.55	0.50	0.99	0.72	0.33	0.28	2.13	1.00
Kcat/Km ($M^{-1}s^{-1}$)	25.58	7.14	8.88	84.24	63.24	7.42	2.19	12.24

Table S10. List of primers used in this study

	Primer name	Sequence (5' to 3')
Primers for reconstructing pMAL-c4x	pMAL-Ml01G0494-F	agggaaggatttcagaattcATGGGATCCCTCAAGAACGC
	pMAL-Ml01G0494-R	aagcttgcctgcaggtcgacCTACTTGGTGAACTCCATGACCC
	pMAL-Ml04G2958-F	agggaaggatttcagaattcATGGCCGGTCCTCACGTG
	pMAL-Ml04G2958-R	aagcttgcctgcaggtcgacCTATTTTGTGAATTCTAGTGCCCAGG
	pMAL-Ml08G0855-F	agggaaggatttcagaattcATGGCATCGGAGAACCAGAA
	pMAL-Ml08G0855-R	aagcttgcctgcaggtcgacTCACTTGATCCTGCGGCAG
	pMAL-F	GTCGACCTGCAGGCAAGCT
	pMAL-R	GAATTCTGAAATCCTTCCCTCGA
Primers for reconstructing pBinPLUS.GFP4	pBin-Ml01G0494-F	cagtgctcagaattacgatcATGGGATCCCTCAAGAACGC
	pBin-Ml01G0494-R	ggcgacattcaaccgattgaCTTGGTGAACTCCATGACCCA
	pBin-Ml04G2958-F	cagtgctcagaattacgatcATGGCCGGTCCTCACGTG
	pBin-Ml04G2958-R	ggcgacattcaaccgattgaTTTTGTGAATTCTAGTGCCCAGG
	pBin-Ml08G0855-F	cagtgctcagaattacgatcATGGCATCGGAGAACCAGAA
	pBin-Ml08G0855-R	ggcgacattcaaccgattgaCTTGATCCTGCGGCAGAGG
	pBin-F	TCAATCGGTTGAATGTCGCC
	pBin-R	GATCGTAATTCTGAGCACTGTCGC

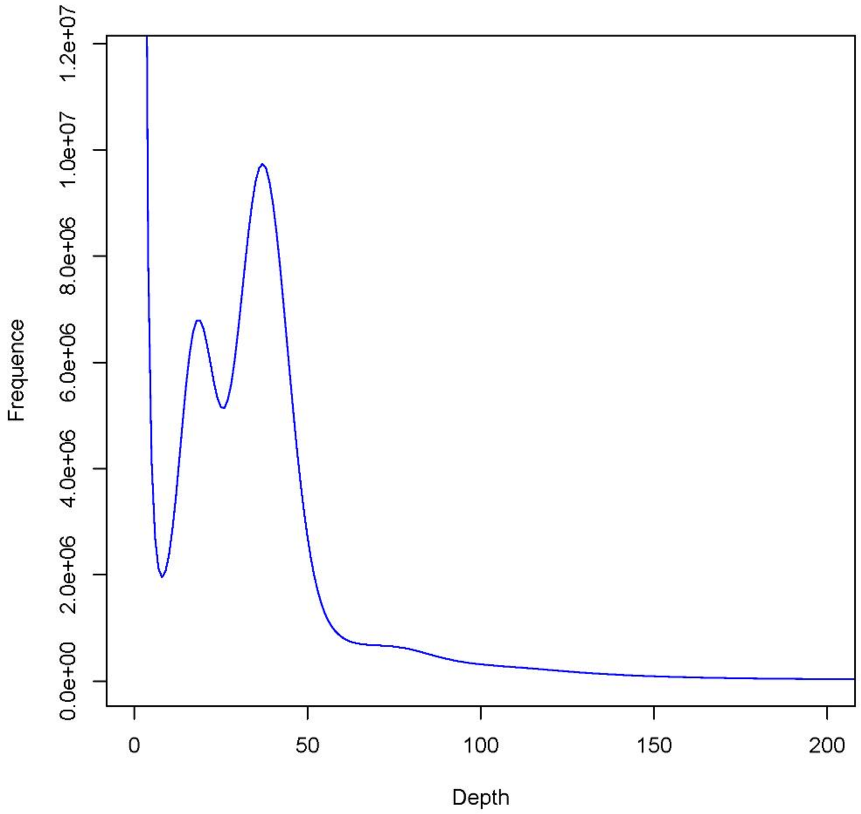

Figure S1. K-mer analysis for evaluating the genome size of *M. lasiocarpa*.

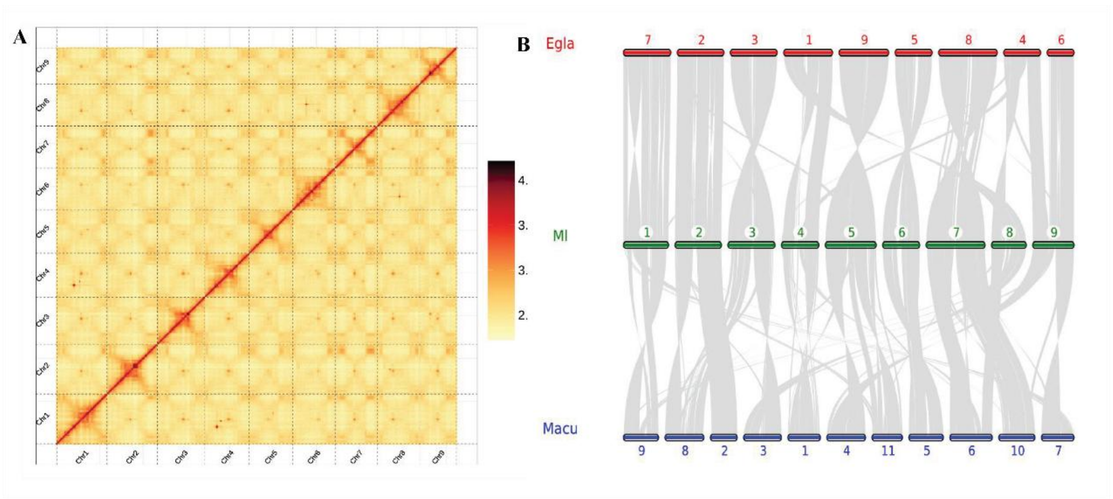

Figure S2. Genome-wide chromatin interactions of nine chromosomes (A) and Genomic collinearity between *M. lasiocarpa* (Ml), *E. glaucum* (Egla), and *M. acuminata* (Macu) (B).

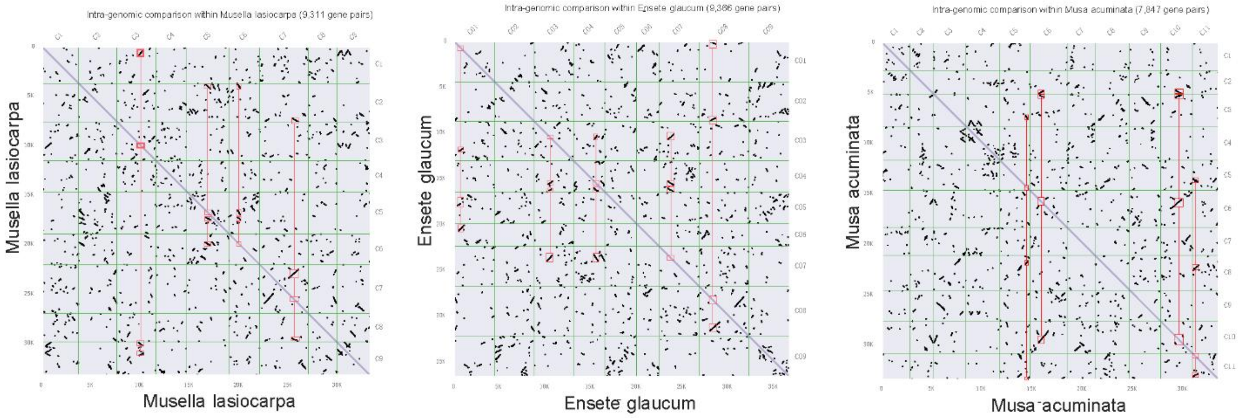

Figure S3. Intragenomic synteny analysis of *Musella lasiocarpa*, *Ensete glaucum*, and *Musa acuminata*.

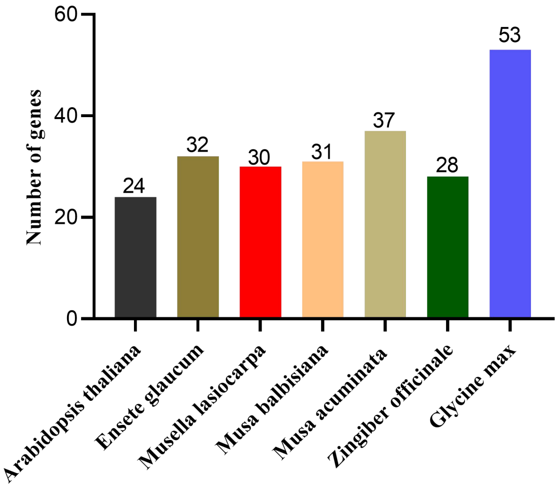

Figure S4. The distribution of *O*-methyl transferase genes in different species which screened by the combination of conserved domains PF01596 or PF00891.

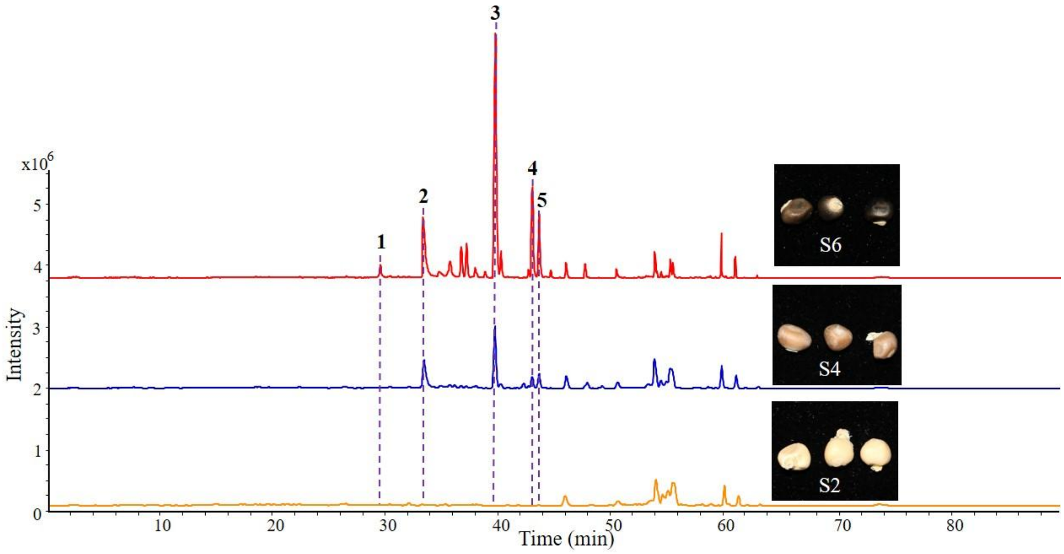

Figure S5. The relative contents of main phenylphenalenone compounds in seeds of different developmental stages by HPLC/Q-TOF MS. **1**, MLT-9; **2**, MLT-2; **3**, MLT-1; **4**, MLT-5; **5**, MLT-3.

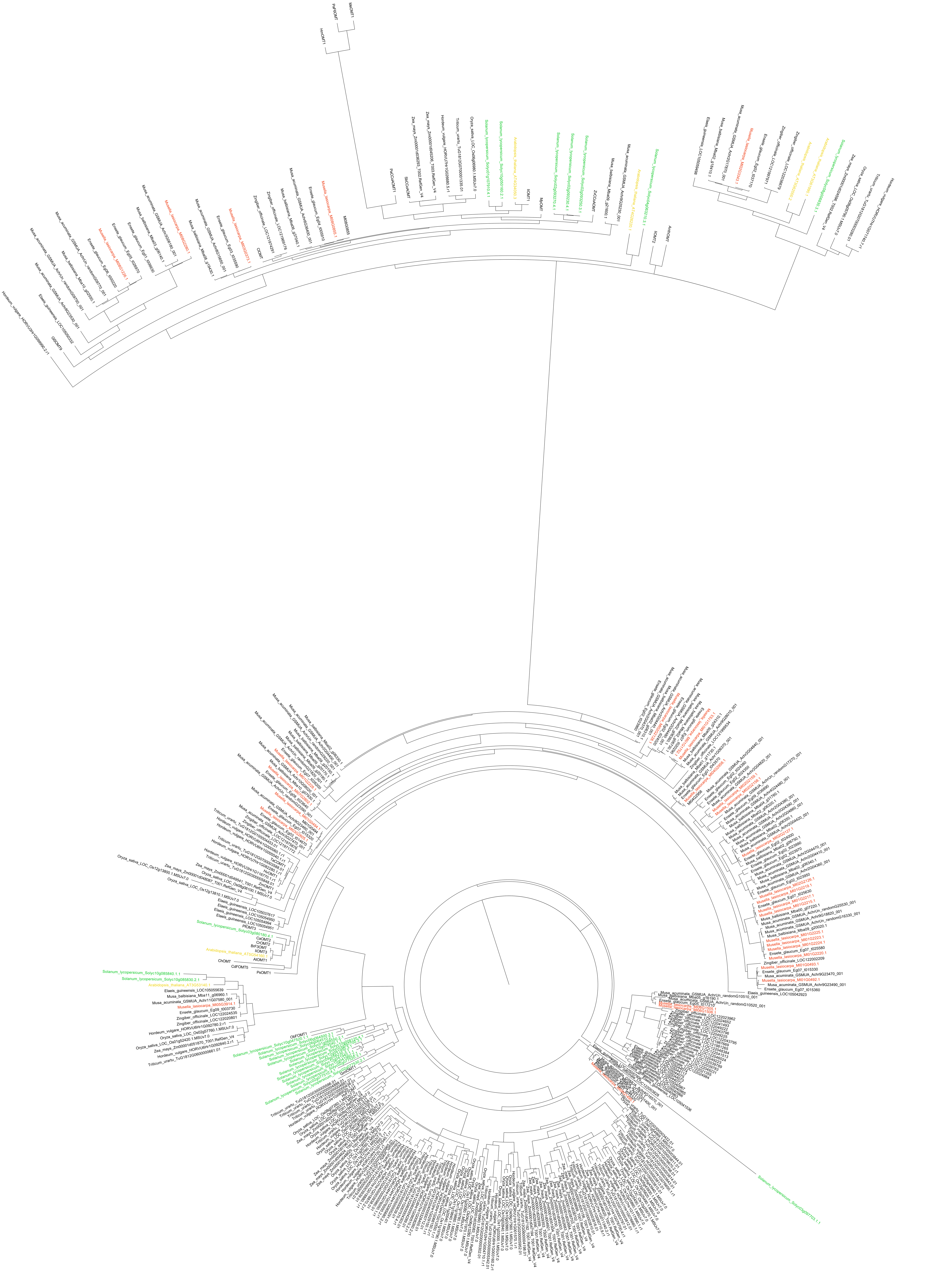

Figure S6. Phylogenetic tree of all OMTs identified from 12 plant genomes.

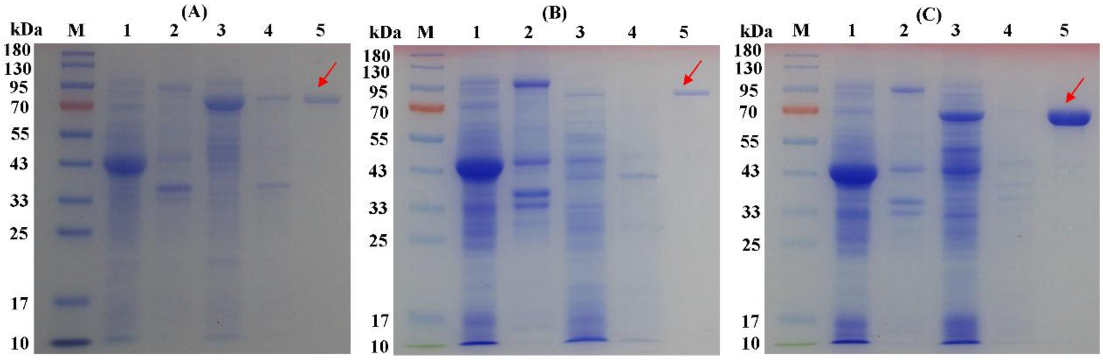

Figure S7. SDS-PAGE of the MlOMTs recombinant proteins. M, marker; (A), Lane 1: the supernatant of empty vector (pMAL-c4x); Lane 2: the pellet of empty vector (pMAL-c4x); Lane 3: the supernatant of Ml01G0494; Lane 4: the pellet of Ml01G0494; Lane 5: purified Ml01G0494; (B), Lane 1: the supernatant of empty vector (pMAL-c4x); Lane 2: the pellet of empty vector (pMAL-c4x); Lane 3: the supernatant of Ml04G2958; Lane 4: the pellet of Ml04G2958; Lane 5: purified Ml04G2958; (C), the supernatant of empty vector (pMAL-c4x); Lane 2: the pellet of empty vector (pMAL-c4x); Lane 3: the supernatant of Ml08G0855; Lane 4: the pellet of Ml08G0855; Lane 5: purified Ml08G0855.

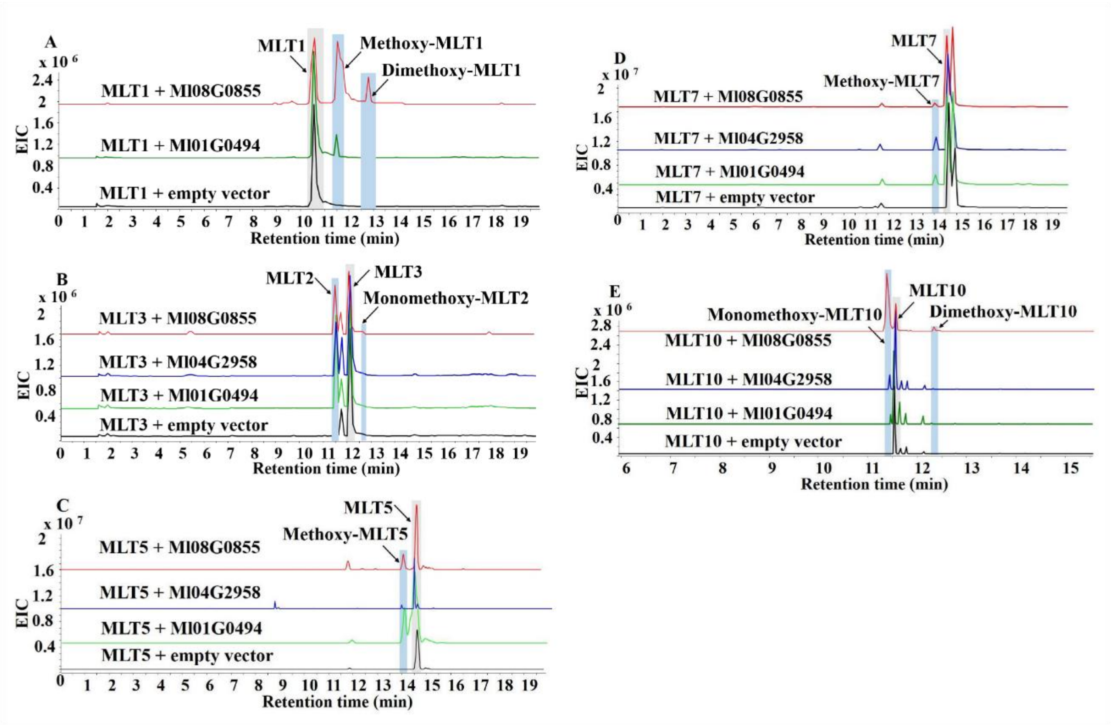

Figure S8. Ml01G0494, Ml04G2958, and Ml08G0855 selectively catalyzed the conversion of substrates in vitro as revealed by HPLC/Q-TOF MS. A, the results of substrate MLT1 reacted with Ml01G0494, and Ml08G0855; B, the results of substrate MLT3 reacted with Ml01G0494, Ml01G2958, and Ml08G0855; C, the results of substrate MLT5 reacted with Ml01G0494, Ml01G2958, and Ml08G0855; D, the results of substrate MLT7 reacted with Ml01G0494, Ml01G2958, and Ml08G0855; E, the results of substrate MLT10 reacted with Ml01G0494, Ml01G2958, and Ml08G0855;

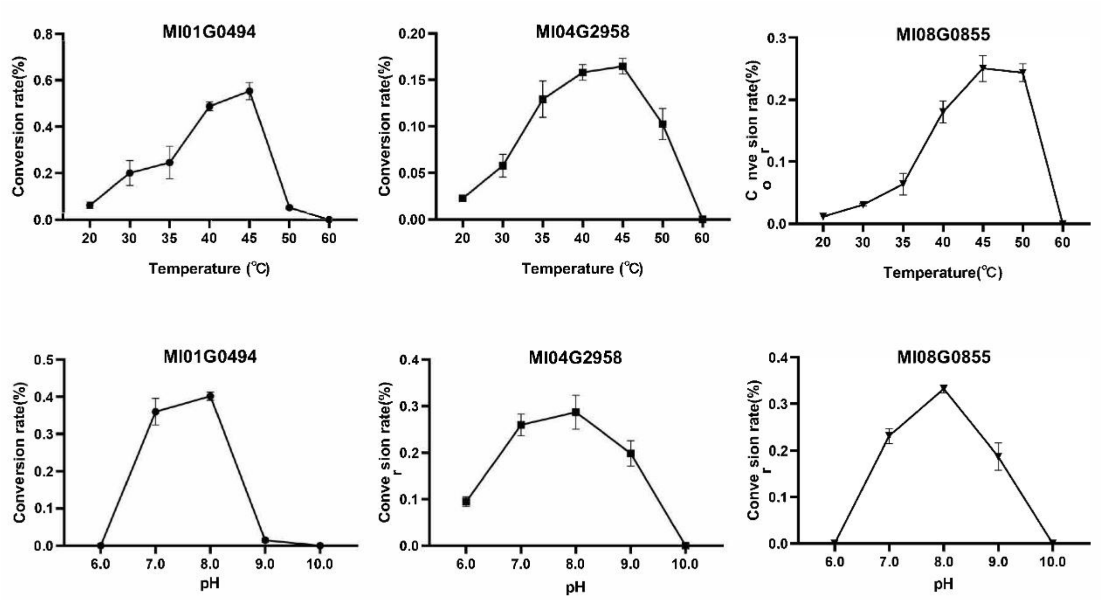

Figure S9. The influence of temperature and pH on the recombinant proteins' activity. MLT4 was used as substrate for assay and the peak area of its methylated product MLT6 was used to determine the relative activity of recombinant proteins, respectively.

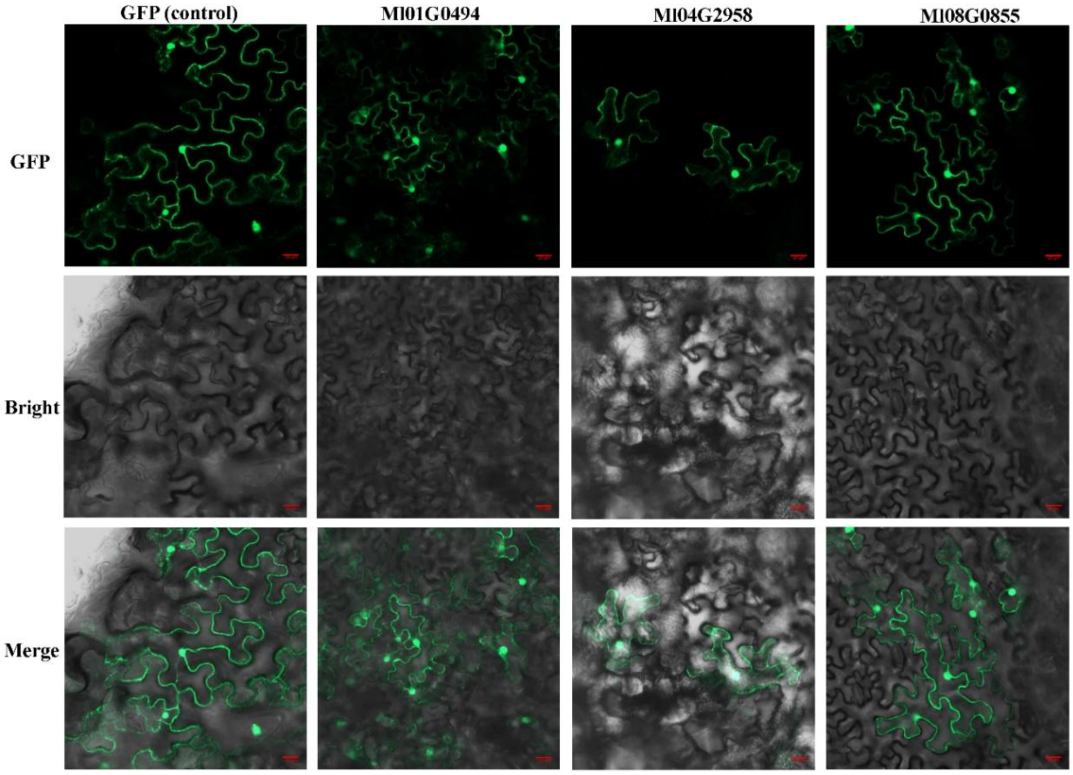

Figure S10. Subcellular localization of MlOMTs with GFP tags in *N. benthamiana*. Scale bar = 20 μm.

Antifungal activity assay of phenylphenalenone compounds

The antifungal activity of campestrol and phenylphenalenone compounds isolated from *M. lasiocarpa* (Figure S12) was determined by the mycelium growth rate method. The strain used for the assays was *Fusarium oxysporum* f. sp. cubense, Foc 4. The compounds were dissolved in DMSO and added to potato sucrose medium to give a final concentration of 50 μg·mL^{-1}. Fungi for assays were taken from the edge of thriving colonies using a 5 mm o.d. borer, and the resulting discs were used to inoculate control and drug-containing plates, respectively. The diameter of the colonies was measured by the cross-crossing method. Inhibition rate = [(diameter of solvent control colony - diameter of drug-treated colony) / diameter of solvent control colony] × 100%. Thiophanate methyl (TM) was used as the positive control, while DMSO alone was used as negative control. Each experiment was carried out in triplicate.

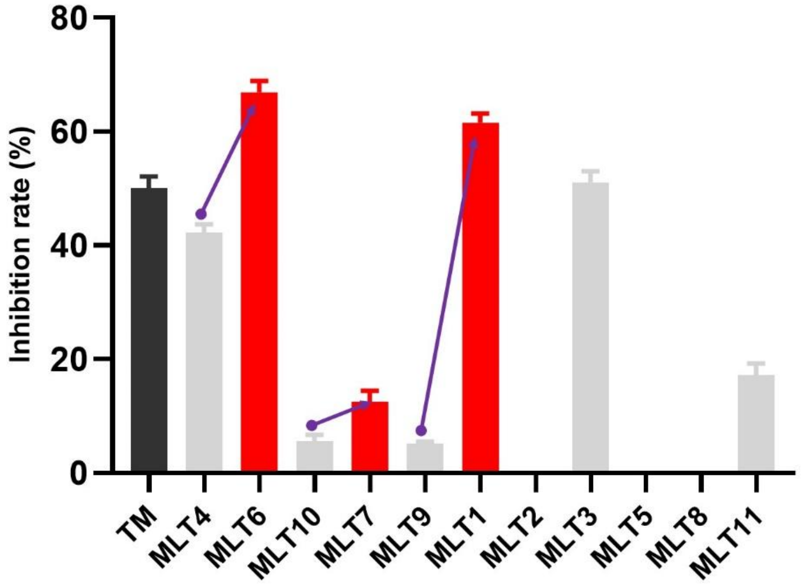

Figure S11. Antifungal activity of phenylphenalenone compounds from *M. lasiocarpa*. When 2-hydroxyl groups were derivatized by methylation in either 4- or 9-PhPNs, the antifungal activity increased.

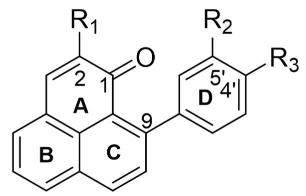

MLT1 R_1 = OCH_3, R_2 = OH, R_3 = OH
MLT2 R_1 = OCH_3, R_2 = H, R_3 = OH
MLT3 R_1 = OH, R_2 = H, R_3 = OH
MLT4 R_1 = OH, R_2 = H, R_3 = H
MLT6 R_1 = OCH_3, R_2 = H, R_3 = H
MLT7 R_1 = OH, R_2 = H, R_3 = OCH_3

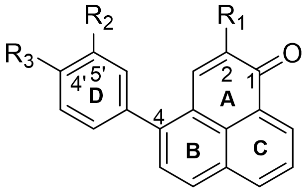

MLT5 R_1 = OH, R_2 = H, R_3 = OCH_3
MLT9 R_1 = OH, R_2 = OH, R_3 = OH
MLT10 R_1 = OH, R_2 = H, R_3 = OH

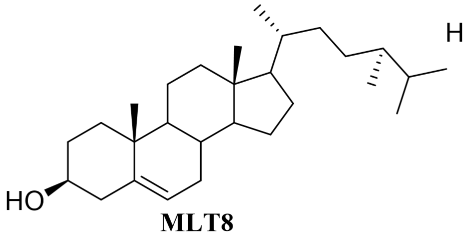

MLT8

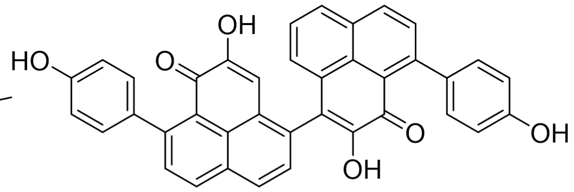

MLT11

Figure S12. The compounds isolated from *M. lasiocarpa*.
2-methoxy-9-(3',4'-dihydroxyphenyl)-1*H*-phenalen-1-one (**MLT1**), 2-methoxy-9-(4'-hydroxyphenyl)-1*H*-phenalen-1-one (**MLT2**), 2-hydroxy-9-(4'-hydroxyphenyl)-1*H*-phenalen-1-one (**MLT3**), 2-hydroxy-9-phenyl-1*H*-phenalen-1-one (**MLT4**), 2-hydroxy-4-(4'-methoxyphenyl)-1*H*-phenalen-1-one (**MLT5**), 2-methoxy-9-phenyl-1*H*-phenalen-1-one (**MLT6**), 2-hydroxy-9-(4'-methoxyphenyl)-1*H*-phenalen-1-one (**MLT7**), campesterol (**MLT8**), 2-hydroxy-4-(3',4'-dihydroxyphenyl)-1*H*-phenalen-1-one (**MLT9**), 2-hydroxy-4-(4'-hydroxyphenyl)-1*H*-phenalen-1-one (**MLT10**), 3,3'-bis-hydroxyanigorufone (**MLT11**).

The Coding DNA Sequence of MlOMTs

>Ml01G0494

ATGGGATCCCTCAAGAACGCGCTGAAGCTGACCCTCGAGGAGGACGAGGACGCGTGC
ATGTACGCCATGCAGCTGGCGAGCGCCTCCATCCTGCCCATGACGCTCAAGGCGGCCA
TCGAGCTGGACCTGCTCGAGATCCTCGTCAGGGCCGGCCCGGGCGCCCGGCTGAGCC
CCGCCGACGTGGTGGCCCAGCTTCATACCGAGAACCCTCAGGCTGCCGTGATGGTGGA
CCGGATGCTCCGCCTGCTCGCCGCCTACAACGTCGTCAGCTGCACCGTCAGCACCGAC
GCCCAAGGGAGGCCATCGAGGAAGTACGGCGCCGCGCCGGCGTGCAAGTACCTGACC
AAGAACGAGGACGGGGTGTCCATGGCCGCTCTGGCCCTGATGAACCAGGACAAGGTC
CTCATGGAGAGCTGGTACTACTTGAAGGACGCGGTGTTGGACGGCGGCATCCCCTTCA
ACAAGGCGTACGGGATGACGGCGTTCGAGTACCACGGCACGGACGCCCGGTTCAACA
AGGTGTTCAACGAGGGCATGAGGAACCACTCCACCATCCTCACCAAGAAGCTGCTCG
ACATCTACCGCGGCTTCGAGGGCGTCAAGGTGCTCGTCGACGTCGGCGGCGGCATCGG
CGCCACCCTCTACATGATCACCGCCAAGCACCCCCACATCAGGGGCATCAACTTCGAC
CTCCCTCACGTCATCTCCGAGGCGCCACCCTTCCCAGGGGTGGAACACGTCGGCGGAG
ACATGTTCGCGAGCGTCCCAAGCGGAGATGCAATCTTCATGAAGTGGATTCTCCATGA
CTGGAGCGACGAGCACTGCGCCAAGATCCTGAAGAACTGCTGCGAGGCGCTGCCGGA
GAAGGGGAAGGTGATAGTGGTGGAGTGCGTCCTGCCCGTGGTTCCGGAGCCGACTCC
CCGAGCCCAGGGCGTCTTCAACATCGACCTCATCATGCTGGCGCACAACCCGGGAGGG
AAAGAGAGGACGGAGAAGGAGTTCGAGGGGTTGGCCAAGGAGGCAGGCTTCTCCGG
ATTCAAAGCGAGCTACATCTTTGCCAACACCTGGGTCATGGAGTTCACCAAGTAG

>Ml04G2958

ATGGCCGGTCCTCACGTGGTTGATGCAGTCGGCGAGCACTCGGAGCCATAT
AAGTCAGCGTCGCGTGCCCACCACCATCACTACCGCCACCACTCATCTCTC
CCCCTGCGAACAATGGGATCCGTGAGGGCGGCGGCGGCGCTGCAACC
GAGCCCGGAGGAGGACGAGGAGGCGTGCTTGCAAGCGGGGCAGCTGGTG
AGCAGTTCCGTCCTCCCCATGACCCTCAAGGCCGCCATCGAGCTCCAGCTC
CTGGAGATCATCGTCGGCGCCGGCCCCGGCGCCAGGCTCAGCCCCGCCGA
CGTTGCGGCCCAGCTGCCCACCACGAACCCGCAGGCGGCCGCCATGGTGG
ACCGCATCCTCCGCCTCCTCGCCGCCTACGGCATCGTCGGCTGCTCCGTCG
AGGCCGGCCCCGACGGCCGCCCTTGCCGCAAGTACGGCGCCGCGCCCGTC
TGCAAGTACCTGACCAGGAACGAGGACGGCGTGTCCTTCGCCGCCCTGAG
CCTGATGAACCAAGACAAGGTCCTCATGGAGAGCTGGTACCACTTGAAGG
ATGCGGTGTTGGAGGGCGGCATCCCCTTCAACAAGGCTTACGGGATGACG
GCGTTCGAGTACCACGGCACCGATCCGCGGTTCAACAAGGTGTTCAACGA
GGGCATGAGAAGCCACTCCATCATCATCACCAAGAAGCTCCTCCAGGTCTA
CCGCGGCTTCGACGACGTCAAGGTGCTCGTCGACGTCGGCGGCGGCACCG
GCGCCACGCTGCACATGATCACCTCCACGCACCCTCACATCCTGGGCATCA
ACTACGACCTCCCTCATGTCATCTCCGACGCGCCGCCCTTGCCAGGCGTGG
AGCATGTCAGCGGCGACATGTTTGAGAGCGTTCCCGGTGGAGACGCCATTC
TTATGAAGTGGATCCTACATGACTGGAGTGATGAGCATTGTGCAAAGATATT
GAAGAACTGTTGGAAGGCTTTGCCAGAGAAGGGAAAGCTGATAGCAGTGG
AATGTGTTCTTCCAGTAGTCCCAGAGCCAAGTCTCAGAACACAGAGTGTTT

GCCATGTAGATCTCATCATGTTGGCTCACAATCCTGGAGGCAAAGAGAGAA
CTGAAATGGAGTTCGAGGAGTTGGCAAAGCAAGCTGGCTTCTCAGGGTTT
AAACCCACTTATGTGTATGCCAGTACCTGGGCACTAGAATTCACAAAATAG

>Ml08G0855
ATGGCATCGGAGAACCAGAACGGAGACCGCAGGCACCAGGAAGTCGGCC
ACAAGAGCCTTCTTCAGAGCGATGCGCTCTATCAGTACATATTGGAGACGA
GCGTGTACCCTCGTGAGCCTGAGGCCATGAAGGAGCTCAGAGAGATCACT
GCCAAACATCCATGGAACTTGATGACCACCTCGGCTGACGAGGGGCAGTT
CCTCAGCATGCTCCTCAAACTGATCAACGCCAAGAACACCATGGAGATCGG
CGTCTACACGGGGTACTCCCTCCTCGCGACCGCCCTCGCGCTCCCCGACGA
CGGCAAGATCCTGGCCATGGACATTAACCGGGAGAACTACGAGATCGGCCT
CCCTGTGATCCAGAAGGCGGGCGTCGCCCACAAGATCGATTTCCACGAAG
GCCCTGCCCTCCCCGTCCTCGATCAAATGATAGAAGATGAGAAGAACCATG
GGTCGTTCGACTTCGTCTTCGTGGACGCGGACAAGGACAACTACATCAACT
ACCACAAGCGACTGCTGGATCTGGTGAGGGTGGGCGGCGTCATCGCCTAC
GACAACACGCTGTGGAGCGGGTCGGTGGTGGCGCCGCCGGACGCCCCCAT
GCGCAAGTACATCCGCTACTACCGGGACTTCGTGCTGGAGCTCAACAAGGC
GCTGGCGGCGGACCCCCGCATCGAGATCTGCCAGCTCCCCGTCGGCGACG
GCGTCACCCTCTGCCGCAGGATCAAGTGA
